# Disease predictability review using common biomarkers appearing in diabetic nephropathy and neurodegeneration of experimental animals

**DOI:** 10.1186/s42826-022-00113-8

**Published:** 2022-02-07

**Authors:** Sun Shin Yi

**Affiliations:** 1 Department of Biomedical Laboratory Science, College of Medical Sciences, Soonchunhyang University, Asan, 31538 Republic of Korea; 2 BK21 Four Program, Department of Medical Science, Soonchunhyang University, Asan, 31538 Republic of Korea

**Keywords:** Cerebro-renal interaction, Chronic kidney disease (CKD), Neurodegenerative disease, Biomarkers, Experimental animal model

## Abstract

It is recently known that the kidney and brain have a very rich distribution of blood vessels, and the histological structures of micro-vessels are very similar. Therefore, a number of studies have reported that renal diseases like chronic kidney disease (CKD) caused by various causes have a very close relationship with the occurrence of neurodegenerative diseases. On the other hand, since diabetic nephropathy, which is caused by chronic inflammation, such as diabetes, often shows very different prognoses even in patients at the same clinical stage, the judgment of their disease prognosis will have a critical meaning in clinical practice. Recently, many studies of cerebro-renal interaction have been reported using experimental animals. The discovery of common biomarkers found in both organs can predict the prognosis of renal disease and the possibility of neurodegenerative disease progression. More associations can be found with novel common biomarkers found in the brain and kidneys that seem entirely unrelated. In that case, it will ultimately be a research field that can expand predictive models of patients' complex diseases through these biomarkers in clinical practice. It is presented biomarkers such as α-klotho, Nephrin, and Synaptopodin. These markers are observed in both the brain and kidney, and it has been reported that both organs show a very significant change in function according to their expression. Even though the brain and kidneys perform very independent functions, it is thought that it has a crucial diagnostic significance that the genes commonly expressed in both organs are functionally effective. With the discovery of novel biomarkers that share cerebro-renal interactions at the early stage of diabetic nephropathy, physicians can predict post-clinical symptoms and prevent severe neurodegenerative and cerebrovascular diseases. Therefore, further study for the diseases of these two organs in laboratory animals means that the field of research on this relationship can be expanded in the future. In the future, more attention and research will be needed on the possibility of prediction for the prevention of neurological diseases caused by CKD in disease animal models.

## Background

Recently, the human life span has increased dramatically by over several decades, and quality of life is considered to be equally important. Diabetes is the most threatening risk factor due to its severe secondary effects on organs such as the kidney, cardiovascular system (CVS), liver, pancreas, and brain. In particular, renal damage, neurological disorders and cerebrovascular disease are strongly associated with diabetes. This cerebro-renal interaction is closely related to microvascular disease and may have a common pathogenesis, since these two organs are connected to each other and have hemodynamic similarities in vascular beds [1]. So far, although research on each kidney disease and degenerative brain disease is active, not many groups seem to focus on the close relationship between the two organs. However, among numerous studies, I would like to introduce three common markers in the diagnosis of diseases and the pathological characteristics of the two organs. Generally, kidney damage markers are albuminuria/proteinuria, serum creatinine and a reduction in estimated glomerular filtration rate (eGFR), which is also a critical marker of CKD in the kidney. In addition, novel biomarkers, such as α-klotho, Nephrin gene (NPHS1), Synaptopodin and so on, for estimating kidney function and renal inflammatory response have been reported in diabetic animal and human studies [1–9].

Many studies have strongly asserted that three biomarkers mentioned above (α-klotho, Nephrin, Synaptopodin) are also available to detect the risks for future cognitive impairment and neurodegenerative disorders early in diabetic animals with renal damage [2, 4–7, 9–12]. Moreover, since the above three markers are found in BBB in the brain [8, 12–16], it is expected to be closely related to the study of dementia caused by kidney disease. Therefore, we consider these two organs to function independently of each other. If either of them fails functionally, it is likely to put our quality of life at severe risk. We believe that introducing three common biomarkers can predict the progressive deterioration of kidney and brain functions. In addition, verification work on its usefulness through discovering additional common biomarkers will have to be continuously added.

## Main text

### Brain and kidney

A clinical relationship has been demonstrated between CKD and cerebrovascular disease/cognitive impairment such as stroke, white matter lesions, silent brain infarction, microhemorrhages, hypertensive nephroangiosclerosis and dementia [1]. Both of the organs involved are low resistance end-organs exposed to high-volume blood flow throughout the cardiac cycles [1]. They have hemodynamic similarities within the endothelial vascular beds in the kidney and the brain [17]. This may reflect shared pathophysiological mechanisms between the two organs. Therefore, vascular damage induced by high blood pressure or diabetes mellitus can occur in both organs and may develop into various disorders by similar mechanisms. Interestingly, patients at the same clinical stage of diabetic nephropathy show diverse prognoses; patients may have the same clinical diabetic phase, but physicians cannot predict their exact future prognosis. Therefore, if the severity could be predicted using clinical samples with novel biomarkers originating from the patients, the diabetic nephropathy can be better managed and neurodegenerative disorders following damage to kidney functions by progressive diabetic nephropathy can be prevented. In particular, many novel biomarkers have been observed in both renal dysfunction and cerebrovascular disorders and several potential candidates have been explored in animal models and human studies. Therefore, the current research focus on finding effective and critical common novel biomarkers with chronological relationships from renal dysfunction to cerebrovascular disorders and cognitive impairments is extremely important to ensure that patients at diverse phases of diabetic nephropathy do not develop other severe diseases such as stroke, neurodegenerative disorders and cognitive impairment.

### Novel prediction biomarkers between brain and kidney

#### α-Klotho

α-*Klotho* was first studied in the construction of the α-*klotho* gene transgenic mouse strain and was initially described as an anti-aging, antioxidant, and cardio-renal protective protein [2, 3, 5, 18]. Recently, two other homologous types of klotho, *β-* and *γ-klotho*, were identified [19]. Here, we only focus on the role of klotho. The gene, in particular, *α-klotho*, has received attention recently due to its involvement in renal and brain functions (Table 1) [3]. α-*Klotho* is known to be expressed principally in important tissues for calcium homeostasis such as distal tubule cells of the kidney, choroid plexus in the brain, and the main cells of the parathyroid gland [34]. Many studies reported that α-*klotho* is a good biomarker for the detection of CVS, brain, and renal dysfunctions: Li et al. [4] reported the immunohistochemical localization of α-*klotho* protein in the brain, kidney, and reproductive organs of mice. α-*Klotho* is not only noted as an anti-aging gene but is also known to be associated profound cardiovascular disease (CVD) [35, 36]. CKD represents extremely low α-*klotho*’s level, suggesting α-*klotho* deficiency may be strongly associated with pathogenesis of CKD-associated CVD [36]. Not only this, in animal experiments or CKD patients, it was confirmed that vascular calcification, cardiac hypertrophy, and uremic vasculopathy were accompanied by α-*klotho* deficiency [18, 23, 24, 37]. In learning and memory tests, α-*klotho* deficiency is remarkably rapid onset the lifespan of mice [5, 22]. The deficiency of α-*klotho* is recognized as a significant clue, especially in the pathogenesis of cognitive impairment [5]. There have been reports that it has a potent effect on CVD and renal failure [38]. Therefore, it can be said that an appropriate α-*klotho* level is a diagnostically critical biomarker for the overall health conditions of the body.

**Table 1:** Biomarker isoforms and functions

	Isoforms	Function	References
α-Klotho	β, γ	• Sensitivity to insulin/anti-aging effects • Bone homeostasis • Suppress oxidative stress, inflammation, endothelial dysfunction • CKD associated thrombosis • Related with hypertension and cardiac hypertrophy	[3, 5, 18–24]
Nephrin (NPHS1)	NPHS2 (Independently called podocin)	• Renal filtration barrier • A transmembrane protein that is a structural component of the slit diaphragm of glomerular podocytes • In defecting, associated congenital nephrotic syndrome, and massive proteinuria • Cardiovascular development • Interaction with glutamate receptors in CNS	[4, 6, 8, 11, 15, 25, 26]
Synaptopodin	-	• Actin-associated protein (actin-based cell morphology and motility) • Regulation of dendritic spine plasticity, synaptic plasticity • Regulation of podocyte cell migration • Associated with serum creatinine level	[8, 9, 27–33]

In addition, Lee et al. [3] reported that soluble *α-klotho* levels in plasma and urine may be a novel and useful early marker of diabetic renal injury. Since *α-klotho* is detected in the plasma and urine, it is expected to be an early marker that is easy to collect from humans and animals for predicting renal injury and neuronal dysfunction in experimental animals and in patients with type 2 diabetes.

#### Nephrin

*Nephrin* gene mutation in congenital nephrotic syndrome (NPHS1) in a mouse model was detected by inactivating NPHS1 in embryonic stem cells by homologous recombination [6]. NPHS2 is independently called podocin, which its mutations that cause severe nephrotic syndrome (Table 1). Putaala et al. reported that *Nephrin* expression is seen in several cerebral regions including the cerebellum, the glomeruli of the main olfactory bulb and the hippocampal dentate gyrus [6]. *Nephrin* is an immunoglobulin-like adhesion molecule first discovered as a major component of the podocyte slit diaphragm, where its integrity is essential to the function of the glomerular filtration barrier. Each podocyte’s cell body has a number of primary processes that extend and further divide, giving rise to interdigitating secondary processes that completely cover the glomerular basement membrane. Due to these morphological characteristics, recent research has emphasized biochemical and functional similarities between podocytes and neuronal cells [7]. The *Nephrin* gene in the rodent model is known to be distributed in various regions within the central nervous system (CNS), and a neurological phenotype was recently reported for *Nephrin*-KO mice after selective rescue of *Nephrin* in the kidney [6]. The precise function of *Nephrin* in the CNS remains to be clarified, yet the gene is expected to be an important biomarker representing the relationship between the kidney and brain in the future.

#### Synaptopodin

As emphasized by continued interest, evidence has been accumulating that neurons and podocytes share a lot of common biological features and morphology [39]. Brain and kidney tissues share common pathological states for some molecules, and ubiquitin carboxy-terminal hydrolase L1 (UCH-L1), a member of the deubiquitylation enzyme family, is a good example expressed specifically in the two organs [8]. Thus, Sun et al. reported that some neuronal iconic proteins may be expressed in podocytes and demonstrated how neuronal iconic proteins are important for the morphology and function of podocytes [8]. *Synaptopodin* is an actin-associated protein of differentiated podocytes that also occurs as part of the post-synaptic densities actin cytoskeleton and is exclusively a related dendritic spines in a subgroup of telencephalic synapses [40]. According to many reports, *Synaptopodin* expression during development correlates with the maturation of neurons and spines [29, 30, 41–43], and essential for spine formation in neurons in the hippocampus [30, 32, 33, 41, 44] and playing role in learning and memory [31, 44, 45]. Many data about *Synaptopodin* strongly implicate in plastic processes at axo-spinous synapses [46]. It has been already discussed that the kidney-specific *Synaptopodin* isoform also regulate stress fiber formation in podocytes by competitive blocking of Smurf1-mediated ubiquitination of RhoA, for proteasomal degradation [28, 47]. It has been reported that the degradation of *Synaptopodin* significantly affects the cytoskeletal changes of the podocytes with the function of the calcium ion channel [26, 48].

This implies that the pathogenesis of podocyte injury shares the same mechanism as nerve injury, and novel therapeutic targets for nephropathy may apply to both neuronal complications and brain impairments.

## Conclusions

Diabetes is a very critical factor for the quality of life in humans. The diabetic condition is the primary cause of negative issues related to decreased eGFR and neuronal dysfunctions such as cognitive impairment and degenerative brain function. Since both organs (podocytes in the kidneys and neurons in the brain) have morphological similarities and share biomarkers such as α-klotho, Nephrin and Synaptopodin, future studies are bound to uncover a number of interesting results leading to therapeutic developments for neurodegenerative diseases and diabetic nephropathy. Many developed countries are experiencing an aging society, and among them, dementia patients due to neurodegeneration along with CKD and CVD are increasing. In particular, the diabetic population is increasing due to Western diet, lack of exercise, and genetic predisposition. It is known that the above CKD, CVD, and dementia and diabetes have a very high correlation. However, functional problems and pathological progression may not occur independently in each organ but are closely related and quietly exacerbated without attention.

In this review, only three biomarkers were introduced, and it was briefly introduced that they provide critical information between the brain and kidney. However, in the future, problems appearing in both organs will have to be viewed from a different perspective than before. In addition to the detailed insight not provided here, more efficient new biomarkers should be introduced through additional research.

## Data Availability

None.

## Abbreviations

CNS: Central nervous system
CKD: Chronic kidney disease
CVS: Cardiovascular system
CVD: Cardiovascular disease
eGFR: Glomerular filtration rate (eGFR
